# Selective nonreporting of 5-min Apgar scores and its safety assessment of out-of-hospital births: a population-based study of United States’ birth data, 2016–2023 a population based study

**DOI:** 10.1016/j.lana.2025.101350

**Published:** 2025-12-27

**Authors:** Amos Grünebaum, Ruth Landau, Frank A. Chervenak

**Affiliations:** a Northwell Health, Zucker School of Medicine, Hempstead, NY, USA; b Virginia Apgar Professor of Anesthesiology, Columbia University Medical Center, New York, NY, USA

**Keywords:** Apgar score, Home birth, Birthing center, Patient safety, Hospital births, Newborn

## Abstract

**Background:**

The safety of out-of-hospital birth in the United States remains contested. A neglected methodological issue is the selective nonreporting of 5-min Apgar scores, which may conceal adverse outcomes and bias safety comparisons. This study examined whether Apgar score missingness differs systematically by birth setting and whether such “informative missingness” alters risk estimates.

**Methods:**

We conducted a population-based analysis of 3,066,021 term, normal-birthweight, midwife-attended singleton births in the United States (2016–2023). Birth settings included hospitals, freestanding birth centers, and planned home births. Missing 5-min Apgar scores were quantified, and deterministic sensitivity analyses modeled the impact of varying assumptions about unrecorded low scores (<4 and <7). Hospital births served as the reference group.

**Findings:**

Five-minute Apgar scores were missing in 0.13% of hospital, 1.9% of birth-center, and 3.1% of home births. Severe compromise (Apgar <4) occurred in 0.17%, 0.20%, and 0.26%, respectively. When half of missing scores were imputed as <4, adjusted odds of severe compromise increased to 7.7 for home and 4.9 for birth-center births vs. hospitals.

**Interpretation:**

This study evaluates documentation integrity of US births. Selective nonreporting of 5-min Apgar scores at out-of-hospital births introduces major bias, distorting apparent safety of out-of-hospital births. Complete and enforceable outcome reporting is essential for scientific validity and ethically sound informed consent.

**Funding:**

None declared.

**Research in context**

**Evidence before this study**

We searched PubMed and Google Scholar from inception to June 1, 2025, for peer-reviewed studies published in English using the search terms “home birth”, “birth center”, “out-of-hospital birth”, “Apgar score”, “missing data”, “neonatal mortality”, and “informative missingness”. Existing research has established that planned home births in the United States are associated with higher rates of adverse neonatal outcomes compared with hospital births. However, comparative safety studies often rely on datasets where Apgar scores are assumed to be missing at random. Previous studies have noted the presence of missing data in birth certificate registries but have not systematically quantified whether this missingness differs by birth setting or modeled its potential to bias safety estimates. There is a lack of large-scale population-based analyses examining the “informative missingness” of Apgar scores specifically in the context of US birth settings.

**Added value of this study**

To our knowledge, this is the first population-based study to systematically analyze the pattern and impact of missing 5-min Apgar scores across US birth settings (hospital, freestanding birth center, and planned home birth) using a dataset of over 3 million midwife-attended births. We identified a substantial disparity in data completeness: 5-min Apgar scores were missing 24-fold more frequently in planned home births compared to hospital births. Using deterministic sensitivity analysis, we demonstrated that even modest assumptions about these missing scores representing adverse outcomes (informative missingness) significantly increase the estimated relative risk of severe neonatal compromise in out-of-hospital settings. This study provides a new methodological framework for evaluating birth safety data that accounts for systematic non-reporting.

**Implications of all the available evidence**

The systematic non-reporting of Apgar scores in out-of-hospital settings compromises the validity of current safety comparisons and undermines informed consent. Evidence suggesting “equivalent safety” for home births may be biased by the exclusion of adverse outcomes that are selectively not recorded. Regulatory bodies should enforce mandatory reporting of neonatal outcomes across all birth settings to ensure data integrity. Future research on birth setting safety must incorporate sensitivity analyses to account for non-random missing data, and clinical counseling for families considering home birth should include transparency about the limitations of current safety data.

## Introduction

According to the American College of Obstetricians and Gynecologists (ACOG) and the American Academy of Pediatrics (AAP), the Apgar score “provides an accepted and convenient method for reporting the status of the newborn infant immediately after birth and the response to resuscitation if it is needed.”^1^ It evaluates five clinical signs: heart rate, respiratory effort, muscle tone, reflex irritability, and color, each scored 0, 1, or 2, with a total ranging from 0 to 10. The 1-min score reflects the infant's condition at birth and the need for immediate intervention, while the 5-min score assesses the infant's response to resuscitation and correlates with long term neonatal outcomes. Scores of 7–10 are generally considered normal, 4–6 moderately depressed, and 0–3 severely depressed.^1^

Neonatal safety refers to the prevention of harm to newborns during the birth process and immediate postnatal period, commonly assessed through standardized measures including Apgar scores, need for resuscitation, admission to neonatal intensive care, and mortality within the first 28 days of life. Planned home birth remains a debated issue in contemporary obstetric care in the US. Advocates emphasize the potential benefits of physiologic birth, reduced intervention rates, and a more personalized and respectful experience. International models, particularly in the Netherlands, the United Kingdom (UK), and parts of Canada, have demonstrated acceptable maternal and neonatal outcomes for carefully selected low-risk pregnancies within integrated maternity care systems,^2^, ^3^, ^4^ while large population-based studies from the United States (US) raise concerns about neonatal safety.^5^, ^6^, ^7^, ^8^, ^9^, ^10^

The American College of Obstetricians and Gynecologists (ACOG) maintains that hospitals and accredited birth centers remain the safest settings for delivery but also acknowledges that planned home birth may be reasonable for select low-risk women.^11^ This position is supported by several US studies showing increased rates of adverse neonatal outcomes, including low Apgar scores, seizures, and perinatal death, in homebirths compared to hospital births.^5^, ^6^, ^7^, ^8^, ^9^, ^10^ In contrast, proponents of homebirth in the US often cite studies suggesting equivalent or even superior outcomes in out-of-hospital births.^12^, ^13^, ^14^, ^15^ The literature presents a divided landscape: one that pits population-level risk assessments with better outcomes in the hospital against the values of maternal autonomy, lower rates of interventions, and natural birth advocacy. In this context, homebirth in the US remains contested, not only in terms of clinical outcomes, but also how evidence of outcomes is generated, interpreted, and how informed consent is provided to those considering a homebirth.

Recent evidence has further highlighted the scale and clinical relevance of this problem. In a 2025 analysis of United States’ birth certificate data, 5-min Apgar scores were documented in 99.88% of hospital births but only 96.9% of planned home births, a difference that could not be explained by random variation or data entry error.^16^ This discrepancy suggested a pattern of *informative missingness*, a form of bias in which the likelihood that data are missing is *per se* related to the underlying outcome, such as poor neonatal condition rather than random omission.^17^^,^^18^ In informative missingness, missing Apgar scores are not evenly distributed but may cluster in clinically compromised cases, thereby concealing adverse outcomes and distorting comparative safety assessments.^18^^,^^19^ These findings raise concerns that unrecorded adverse neonatal outcomes may be systematically excluded from public data sources, thereby artificially narrowing the apparent safety gap between hospital and homebirth settings.

We hypothesized that missing 5-min Apgar scores are not randomly distributed but represent informative missingness associated with adverse neonatal events. Informative missingness occurs when the probability of data being missing is related to the unobserved value itself, biasing estimates away from less favorable outcomes. This study aims to: (1) quantify the prevalence of missing 5-min Apgar scores across United States’ birth settings among term, normal birthweight infants attended by midwives in hospitals, freestanding birth centers, and planned home births; (2) assess whether missingness rates differ systematically by birth location; and (3) model the potential impact of informative missingness on neonatal risk estimates using sensitivity analysis. The primary outcome was the rate of missing 5-min Apgar scores, with secondary outcomes including rates of low Apgar scores (<4 indicating severe compromise; <7 indicating moderate compromise) under varying assumptions about missing data.

## Methods

### Study design and population

This is a retrospective population-based analysis using US Centers for Disease Control and Prevention (CDC) natality data from 2016 to 2023.^20^ The CDC natality database captures >99% of all United States' births through legally mandated birth certificate reporting across all 50 states and jurisdictions. Standardized variables of US birth certificates include maternal demographics, prenatal care, pregnancy history, labor and delivery characteristics, infant status at birth, and congenital anomalies. These publicly available data are derived from birth certificates, which are legally required for each birth in all 50 states, and include standardized information on maternal demographics, prenatal care, labor and delivery, and neonatal outcomes for nearly all US births. Data completion is mandated by state laws and regulations, following national standards established by the CDC's National Center for Health Statistics (NCHS), which coordinates data collection and quality through the National Vital Statistics System.^20^^,^^21^

Birth certificate data are completed by the birth attendant (midwife or physician) and facility administrative staff, with legal responsibility for accuracy resting with the attendant of record. In hospital settings, data entry is typically verified through quality assurance processes; in out-of-hospital settings such oversight is variable or absent.

### Inclusion and exclusion criteria

#### Study population

Term singleton infants (≥37 weeks’ gestation) with normal birthweight (≥2500 *g*) where the birthplace was listed as either hospital, freestanding birth center, or intended (planned) home birth and who were attended by a midwife (certified nurse midwife or other midwife. “Other midwife” refers to midwives who are not Certified Nurse-Midwives (CNMs) or Certified Midwives (CMs)–primarily Certified Professional Midwives (CPMs), Licensed Midwives (LMs), or lay midwives, depending on state licensure).

#### Exclusions

We excluded all births <37 weeks’ gestation, birthweight <2500 *g*, multiple gestations, births attended by physicians only, and births in settings other than the three specified locations (hospital, freestanding birth center, or intended home birth).

#### Rationale for population restriction

We restricted our analysis to term (≥37 weeks) infants with normal birthweight (≥2500 *g*) which creates an appropriate comparison group and aligns with prior comparative studies of birth setting safety: (1) This represents the population for which out-of-hospital birth is considered clinically appropriate by professional guidelines^11^; (2) Preterm and low birthweight infants have higher baseline rates of both low Apgar scores and missing data due to clinical complexity, which would confound our ability to isolate the effect of birth setting on documentation practices; (3) By examining a homogeneous, low-risk cohort where Apgar scoring should be straightforward, we can more clearly assess whether documentation completeness differs by setting independent of clinical factors; (4) If systematic documentation failures exist in a low-risk population, where complete recording should be most feasible, they likely extend to higher-risk groups as well.

### Birth settings and provider types

In the United States, midwife credentials and licensing vary across states. Certified Nurse-Midwives (CNMs) hold nursing and graduate midwifery degrees and practice primarily in hospitals. Certified Professional Midwives (CPMs) are credentialed through a separate pathway without nursing requirements and practice primarily in homes and birth centers. Hospital births occur in facilities with immediate access to obstetric, anesthetic, and neonatal resuscitation resources. Freestanding birth centers are licensed facilities separate from hospitals, with variable proximity to emergency services. Planned home births occur in private residences with equipment and personnel determined by the attending midwife. Neither freestanding birth centers nor home birth settings are legally required to maintain the full complement of neonatal resuscitation equipment mandated in hospital delivery rooms, including mechanical ventilation, emergency medications, and immediate access to neonatal intensive care. Regulatory standards for out-of-hospital settings vary by state, and compliance is inconsistently monitored.

#### Apgar score documentation practices by setting

Apgar scoring is typically performed by the birth attendant or designee present at delivery at 1-min, 5-min, and occasionally 10-min after delivery. In hospital settings, this may be the delivering physician or midwife, a labor nurse, or neonatal team member, depending on institutional protocols. In hospitals, documentation is typically verified through quality assurance processes. In out-of-hospital settings, the attending midwife is responsible for both performing and recording the Apgar score, with limited external oversight. Birth certificate data do not specify which individual performed the assessment.

#### Apgar score differences across settings

Apgar score documentation standards differ substantially by setting. Hospitals have: (1) multiple personnel present who can perform assessment while others provide care; (2) standardized forms and electronic health records with required fields; (3) quality assurance audits; (4) regulatory oversight from Joint Commission and state health departments. Out-of-hospital settings typically have: (1) only 1–2 providers for both maternal and neonatal care; (2) paper-based documentation completed after the birth; (3) variable oversight depending on state licensing requirements; (4) no standardized quality review.

### Outcome measures

The primary outcome was the rate of missing 5-min Apgar scores (missingness), defined as 5-min Apgar scores recorded as “unknown” or “not stated.” Secondary outcomes included the rates of low Apgar scores, categorized into 2 groups: 5-min Apgar scores <7 and <4. Missingness rates were calculated for multiple clinical and demographic variables across birth settings to identify systematic differences in data completeness and documentation practices. The 5-min Apgar score was selected as the primary outcome because the 1-min Apgar score is not documented in the CDC natality database. In addition, the 5-min score is more predictive of subsequent neurological outcome than the 1-min score and serves as a proxy for perinatal asphyxia and adequacy of resuscitation. 5-min Apgar scores <4 indicate severe compromise requiring intensive resuscitation; scores <7 indicate moderate compromise. Both thresholds are associated with increased neonatal morbidity and mortality.^22^, ^23^, ^24^, ^25^, ^26^, ^27^, ^28^, ^29^, ^30^, ^31^ In addition to the 5-min Apgar score we also examined the following variables for missingness: delivery mode, fetal presentation, infant living, congenital anomalies, abnormal conditions.

### Sensitivity analysis approach

We modeled five scenarios in which 0% (“the baseline”), 25%, 50%, 75%, or 100% of missing 5-min Apgar scores represented low values (<4 or <7). Variables used in imputation scenarios: For each birth setting, we took the observed number of missing Apgar scores and imputed them as low scores (<4 or <7) according to each scenario assumption. For example, in the 25% scenario, we assumed that 25% of the missing scores in each setting would have been <4 (or <7) and added these imputed cases to the observed counts of low Apgar scores. We then recalculated incidence rates and odds ratios for each setting under each scenario.

This approach, considered a methodological standard for evaluating sensitivity to missingness, enabled us to quantify the extent to which incomplete documentation may distort risk estimates across birth settings.^32^, ^33^, ^34^

### Statistical analysis

We used deterministic sensitivity analysis to model five fixed scenarios in which 0%, 25%, 50%, 75%, or 100% of missing 5-min Apgar scores represented values <4 or <7. This approach differs from traditional probabilistic Monte Carlo methods that sample from probability distributions. We selected deterministic scenarios because: (1) we have no empirical basis for assigning probability distributions to unobserved data; (2) fixed scenarios allow transparent interpretation of how different assumptions affect results; and (3) this approach is standard in sensitivity analysis for missing data in observational studies.

How the dataset contributes to the modeling: The observed data provide three essential inputs: (1) the rate of missingness in each birth setting (e.g., 3.12% in home births, 0.13% in hospital births); (2) the distribution of documented Apgar scores among non-missing cases, which establishes baseline risk; and (3) denominators for calculating population-level incidence rates. The sensitivity analysis combines these observed quantities with varying assumptions about unobserved values to model the range of plausible true incidence rates under different levels of informative missingness.

For each scenario, simulated cases were added to observed low Apgar counts in each birth setting. Proportions and odds ratios were recalculated for each scenario using 2 × 2 tables with hospital births as the reference group. A 95% confidence interval was calculated for the observed (non-imputed) data to reflect true sampling variability. The deterministic scenarios represent fixed imputations based on explicit assumptions rather than stochastic estimates with sampling variability; thus, confidence intervals were not computed for these scenarios.

### Rationale for sensitivity analysis method

While both longitudinal models and Monte Carlo simulations are accepted methods for addressing missing data, we selected deterministic sensitivity analysis because our dataset was cross-sectional and lacked the repeated measures required for longitudinal modeling.^32^^,^^33^ Our objective was not to assess within-subject change over time, but to evaluate how informative missingness might bias population-level outcome estimates. Deterministic sensitivity methods allow flexible, assumption-based modeling of unobserved data distributions—making them particularly well suited to large datasets where missing outcome data are likely non-random and potentially conceal adverse events.

## Results

### Apgar scores across birth settings and patterns of missing data

Among term (≥37 weeks gestation), normal birthweight (≥2500 *g*) singleton infants attended by midwives, between 2016 and 2023, there were a total of 3,066,021 births. Of these, births were in hospitals (n = 2,677,875; 87.3%), freestanding birth centers (n = 155,127; 5.1%), and planned homebirths (n = 233,019; 7.6%) (Table 1).

**Table 1:** Distribution of 5-min Apgar scores by birth setting.

5-min Apgar score	Hospital (n = 2,679,689)	Freestanding birth center (n = 155,555)	Home (n = 234,717)
Unknown/Not stated	3397 (0.13%)	2942 (1.89%)	7303 (3.11%)
0	485 (0.02%)	37 (0.02%)	72 (0.03%)
1	765 (0.03%)	37 (0.02%)	103 (0.04%)
2	1423 (0.05%)	98 (0.06%)	165 (0.07%)
3	1836 (0.07%)	133 (0.09%)	257 (0.11%)
4	2923 (0.11%)	229 (0.15%)	427 (0.18%)
5	5731 (0.21%)	470 (0.3%)	1041 (0.44%)
6	12,037 (0.45%)	1028 (0.66%)	1690 (0.72%)
7	35,022 (1.31%)	3042 (1.96%)	4706 (2.0%)
8	242,335 (9.04%)	13,180 (8.47%)	18,802 (8.01%)
9	2,329,618 (86.94%)	98,722 (63.46%)	108,128 (46.07%)
10	44,017 (1.64%)	35,637 (22.91%)	92,023 (39.21%)

Data presented as percentage (absolute number). Apgar scores range from 0 to 10, with scores ≥7 generally considered normal. Unknown/Not Stated category includes cases where 5-min Apgar scores were not documented or recorded. Sample sizes: Hospital births n = 2,679,689; Freestanding Birth Center births n = 155,555; Home births n = 234,717.

**Fig. 1:**
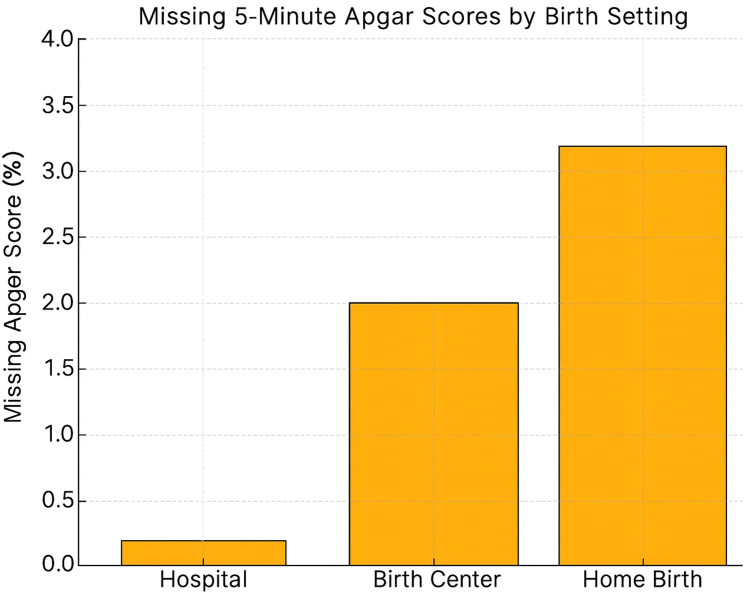
**Missing 5-min Apgar scores by birth setting, United States, 2016**–**2023**. Proportion of midwife-attended term births with missing 5-min Apgar scores, based on United States natality data. Missingness was substantially higher in out-of-hospital settings—3.12% in planned home births and 1.90% in freestanding birth centers—compared to 0.13% in hospital births.

The distribution of 5-min Apgar scores varied significantly by birth setting (Table 1). Hospital births had the lowest proportion of missing scores (0.13%), whereas home births had the highest rate of missing data (3.1%). Notably, 5-min Apgar scores of 10 were recorded far more frequently in out-of-hospital settings (39.2% of home births, 22.9% of birth center births) compared to hospital births (1.6%) (Fig. 1).

**Fig. 2:**
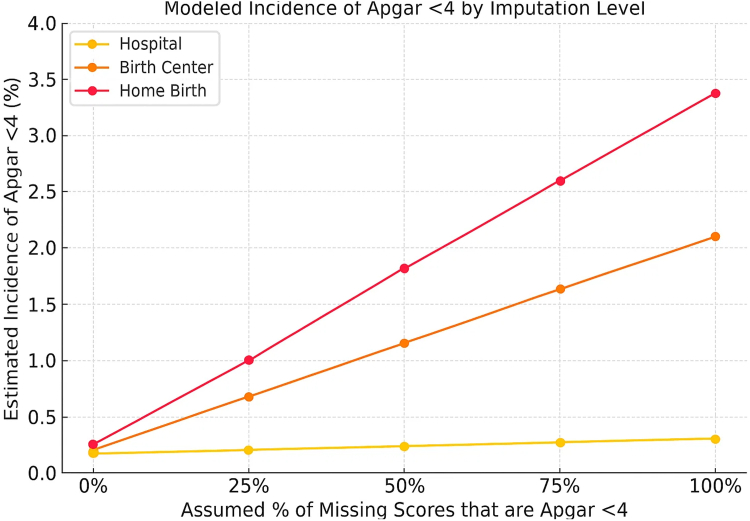
**Modeled incidence of Apgar <4 by birth setting and imputation level, United States, 2016**–**2023**. Deterministic scenario sensitivity analysis estimating the absolute incidence of Apgar <4 under varying assumptions that missing 5-min Apgar scores reflect adverse outcomes. Incidence remains stable in hospital births due to complete documentation, while estimated risk rises substantially in home and birth center settings—reaching 3.38% in home births under full imputation—highlighting the scale of potentially hidden neonatal compromise.

**Fig. 3:**
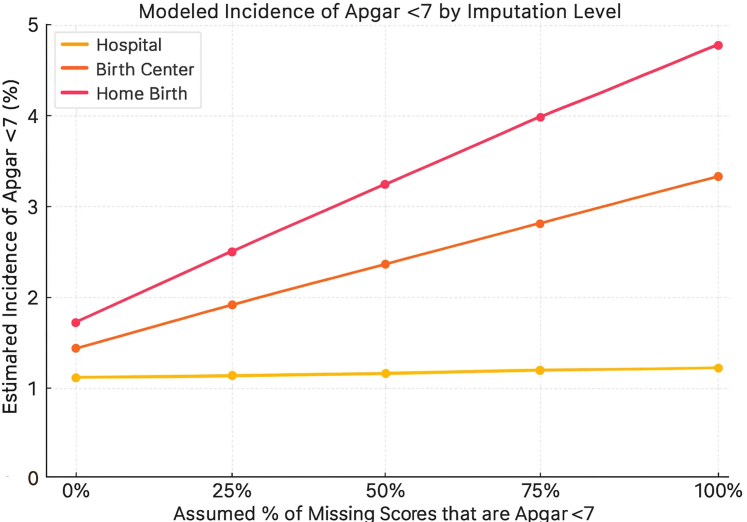
**Modeled incidence of Apgar <7 by birth setting and imputation level, United States, 2016**–**2023**. Deterministic scenario sensitivity analysis estimating absolute incidence of Apgar <7 under increasing assumptions that missing scores reflect adverse outcomes. Home and birth center settings show rising incidence with greater imputation, reaching 4.77% and 3.24% respectively at 100% imputation, while hospital rates remain nearly unchanged—illustrating how missing data may conceal significant differences in neonatal risk.

Table 2 shows additional missing data cross several required variables in hospital and out-of-hospital births. The 5-min Apgar score was missing far more frequently than any other variable. The 5-min Apgar score was missing in 3.12% of planned home births and 1.90% of freestanding birth center births, compared to just 0.13% of hospital births. This represents a 25-fold increased likelihood of missingness in home births (OR 25.4; 95% CI: 24.4–26.4) and a 15-fold increase in birth centers (OR 15.2; 95% CI: 14.5–16.0) relative to hospitals (Table 2). In contrast, other clinical and demographic select variables were more completely documented, even in out-of-hospital settings. This discrepancy was observed across both planned home births and freestanding birth center births. Figs. 2 and 3 show the modeled incidence of Apgar <4 and <7 by birth setting and imputation level.

**Table 2:** Rates and odds ratios of missing data including 5-min Apgar scores by birth setting in United States midwife-attended term births, 2016–2023.

Variable	Hospital	Birth center	Home	Birth center	Home
	(n = 2,678,636)	(n = 155,020)	(n = 233,920)	OR (95% CI)	OR (95% CI)
Delivery mode	595 (0.02%)	12 (0.01%)	766 (0.33%)	0.50 (0.28–0.89)	16.50 (15.23–17.88)
Fetal presentation	3067 (0.11%)	1195 (0.77%)	1722 (0.74%)	7.00 (6.57–7.46)	6.73 (6.38–7.10)
Infant living	1617 (0.06%)	121 (0.08%)	357 (0.15%)	1.33 (1.10–1.61)	2.50 (2.23–2.80)
Congenital anomalies	3222 (0.12%)	658 (0.42%)	1297 (0.55%)	3.56 (3.27–3.88)	4.68 (4.41–4.97)
Abnormal conditions	2812 (0.10%)	659 (0.43%)	1525 (0.65%)	4.19 (3.85–4.56)	6.37 (6.01–6.75)
5-min Apgar score	**3397 (0.13%)**	**2942 (1.90%)**	**7303 (3.12%)**	**15.24 (14.50–16.01)**	**25.38 (24.36–26.44)**

Odds ratios (ORs) and 95% confidence intervals (CIs) were derived from 2 × 2 contingency tables comparing each out-of-hospital setting to hospital births as the reference group. The analysis included term (≥37 weeks), normal birthweight (≥2500 g) births attended by midwives from 2016 to 2023.

To address potential geographic heterogeneity, we examined patterns of missing 5-min Apgar scores across all 50 US states and DC. CDC WONDER's natality database suppresses “small cells” to protect patient confidentiality: when counts fall below a dataset-specific threshold, rows with zero or suppressed births are hidden, and totals or percentage-of-total fields may also be withheld if any contributing cell is suppressed. Suppression policies vary by dataset and level of aggregation, so using larger populations, longer time spans, or broader geographies reduces suppression, and aggregate totals include suppressed values only when permitted by the privacy rules. Therefore, some data for many smaller US states could not be calculated reliably because counts fell below the threshold. The available data show that in each state the same pattern of increased missingness of 5-min Apgar score in planned home births compared to hospital births persisted with no US state showing higher missingness of 5-min Apgar scores in hospital compared to home settings.

### Sensitivity analysis results

For this cohort of term, normal birthweight infants, due to substantial differences in missing 5-min Apgar scores across birth settings, we used deterministic sensitivity analysis to model how risk estimates varied under different assumptions (Table 3). At baseline, before imputing missing data, the incidence of 5-min Apgar scores <4 was significantly higher in-home births (0.26%, OR 1.53; 95% CI: 1.40–1.66) and in birth center births (0.20%, OR 1.18; 95% CI: 1.04–1.32) compared to hospital births (0.17%, reference) (Table 3). For 5-min Apgar scores <7, baseline rates were also significantly elevated in home births (1.65%, OR 1.78; 95% CI: 1.71–1.83) and in birth centers (1.33%, OR 1.42; 95% CI: 1.36–1.49), relative to hospitals (0.94%, reference) (Table 3).

**Table 3:** Sensitivity analysis: odds ratios for 5-min Apgar score <4 and <7 under missing data assumptions.

Missing data assumption	Apgar score <4		Apgar score <7	
	Birth center OR (%)	Home birth OR (%)	Birth center OR (%)	Home birth OR (%)
Baseline (observed)	1.18 (0.20%)	1.53 (0.26%)	1.42 (1.33%)	1.78 (1.65%)
25% = Adverse	3.37	5.16	1.94	2.61
50% = Adverse	5.71	8.21	2.52	3.45
75% = Adverse	7.99	10.70	3.08	4.39
100% = Adverse	10.12	12.30	3.63	5.35

Hospital births = reference group (OR 1.0); Hospital baseline rate: 0.17% (Apgar <4). 0.94% (Apgar <7).

Deterministic scenario sensitivity analysis were used to estimate adjusted rates and odds ratios (ORs) of low 5-min Apgar scores under five scenarios assuming that 0%, 25%, 50%, 75%, or 100% of missing scores represented adverse outcomes (5-min Apgar <4 or <7). Simulated cases were added to observed low Apgar counts in each birth setting. Proportions and ORs were recalculated for each scenario using 2 × 2 tables with hospital births as the reference group. The analysis included all term (≥37 weeks), midwife-attended births from 2016 to 2023 with normal birthweight (≥2500 *g*). A 95% confidence interval was calculated for the observed (non-imputed) data to reflect true sampling variability.

Using hospital births as the reference (OR = 1), imputing that 25% of missing scores were <4 increased the incidence of 5-min Apgar scores in home births from 0.26% to 1.0% (OR 5.16) and in birth centers from 0.20% to 0.68% (OR 3.37) (Table 3). At 50% imputation, ORs rose to 8.21 and 5.71, respectively. Patterns for Apgar <7 were similar: The incidence of low Apgar scores in home births OR rose from 1.78 at baseline to 2.61 (25% imputation) and 3.45 (50% imputation); birth center OR rose from 1.42 to 1.94 and 2.52 (Table 3). Hospital estimates remained stable due to near-complete documentation.

## Discussion

This study addresses a fundamental methodological question: are mandated 5-min Apgar score data documented completely and equally across birth settings? Our findings demonstrate they are not. Understanding the safety of out-of-hospital birth in the US requires not only comparing clinical outcomes, but also ensuring those outcomes are reliably measured and transparently reported. We show that 5-min Apgar scores are disproportionately missing in planned home and birth center settings, suggesting systematic underreporting. Informative missingness may conceal adverse neonatal events and undermine the validity of comparative research.

First introduced by Virginia Apgar in 1953 as a standardized assessment of newborn condition, the Apgar score remains one of the most widely used indicators of neonatal status.^22^ Low 5-min Apgar scores, defined as <4 (severe compromise) or <7 (moderate compromise), are robustly associated in the literature with neonatal mortality and morbidity. Multiple population-based studies demonstrate these associations: Moster et al. reported a 50-fold increased risk of neonatal death with 5-min Apgar <4 vs. 7–10^28^; Thorngren-Jerneck found low scores predicted NICU admission and mechanical ventilation^25^; and Iliodromiti et al. demonstrated long-term effects on cognitive function in adolescence.^27^ The 5-min score is the most predictive of subsequent clinical problems.^23^, ^24^, ^25^, ^26^, ^27^, ^28^, ^29^, ^30^, ^31^

The 5-min Apgar scores were disproportionately missing in out-of-hospital births while demographic and clinical variables, maternal age, race, infant plurality and sex, were nearly universally documented across all settings. This selective absence suggests *informative missingness*, where missing data is linked to adverse events.^32^, ^33^, ^34^ When key indicators are missing in settings where other documentation is complete, it compromises both safety comparisons and informed consent. Even modest assumptions about missing data magnified estimated risk in out-of-hospital settings, while hospital estimates remained stable due to near-complete documentation.

### Patterns suggesting selective reporting and score inflation

The implausibly high rates of perfect Apgar scores (10/10) in out-of-hospital settings reported here and previously also described^35^ warrant attention. A 5-min Apgar of 10 requires perfect scores in all five categories, including complete absence of cyanosis, rare even in healthy term infants. Rates of 39% (home) and 23% (birth centers) vs. 1.6% (hospitals) suggest scoring bias, whether from subjective interpretation, lack of standardized training, or intentional inflation to avoid triggering reporting or transfer protocols. This pattern, combined with higher rates of missing scores, suggests that data quality concerns extend beyond omission to potentially include distortion of recorded values.

### Birth setting as the primary determinant

This study introduces a framework for detecting informative missingness in perinatal research, using over 3 million midwife-attended births. Deterministic sensitivity analyses simulate the impact of unrecorded harm across plausible scenarios, applicable to any outcome where missingness is non-random. Importantly, 5-min Apgar scores were missing at significantly different rates: 3.12% of 5-min Apgar scores were missing in home births and 1.9% in birth centers vs. 0.13% in hospitals, despite near-complete reporting of other variables across settings. This pattern was consistent across all 50 states and all 8 years examined (2016–2023), demonstrating a systematic, national phenomenon rather than isolated problems in specific locations. This discrepancy strengthens the case for systematic underreporting.

Our findings suggest that birth location is the critical factor. It is important to remember that almost 90% of midwife births occur in hospitals, where there is no evidence of informative missingness. The same low-risk population experiences dramatically different documentation rates depending on where birth occurs. Near-complete documentation in hospitals (99.87%) suggests that setting-level factors, institutional protocols, quality oversight, and support staff availability, are important contributors to chart completeness This reflects multiple systems factors: dedicated personnel for documentation during emergencies; institutional quality assurance; regulatory oversight and accountability; and standardized recording protocols. Out-of-hospital settings differ not only in location and resuscitation equipment but also in personnel availability. Hospitals have dedicated nurses, respiratory therapists, obstetricians, and neonatologists who can focus on the neonate while the birth attendant manages the mother. In home and birth center settings, typically only 1–2 midwives manage both patients.

### Quality of binomial (mother-neonate) care by birth setting

Birth setting safety depends on the facility's capacity to simultaneously support maternal and neonatal needs. Hospital labor units are designed with dedicated teams, immediate access to anesthesia and surgery for maternal complications, and neonatal intensive care for infant compromise. Out-of-hospital settings rely on small teams managing both with limited backup. When complications arise, documentation integrity may be compromised regardless of provider skill.

The pattern of missing 5-min Apgar scores aligns with the “Missing Not at Random” framework, where missingness probability depends on the unrecorded value itself. Missing Not at Random occurs when missingness is associated with the value that would have been observed, such that the missing data mechanism cannot be explained by measured covariates alone: low scores are preferentially missing because they are low. This bias cannot be corrected with conventional statistical methods, but deterministic sensitivity analysis offers a transparent way to model its impact. Often quoted US studies supporting homebirth safety reported 2–5% missing Apgar scores without addressing bias,^12^, ^13^, ^14^, ^15^ while others failed to report missingness at all.^36^, ^37^, ^38^, ^39^, ^40^ In contrast, the *Birthplace in England* study and Scottish national data exhibit exemplary completeness.^2^^,^^27^

Contrary to the US out-of-hospital system, the UK systems operate within an integrated national maternity care system with mandatory outcome reporting, standardized midwifery education, and centralized data oversight. In contrast, the US out-of-hospital birth system is fragmented, lacking a national registry, uniform credentialing, enforceable risk exclusion criteria, or standardized outcome reporting. Most midwives attending home births lack formal hospital integration and operate without physician oversight. The Midwives Alliance of North America (MANA), which promoted the Certified Professional Midwife (CPM) credential, recently folded, further weakening oversight. CPMs are not required to hold nursing degrees or meet hospital-based training requirements. Across multiple studies, neonatal mortality was significantly higher in out-of-hospital births attended by CPMs compared to Certified Nurse-Midwives (CNMs).^41^^,^^42^ There are no national guidelines requiring data submission or accountability for adverse outcomes, and emergency service coordination is inconsistent. While the CDC mandates complete birth data, it has no enforcement authority, leaving documentation vulnerable to omission or manipulation.

### Provider behavior vs. infant health: a false dichotomy

A key question is whether missing Apgar scores reflect provider characteristics (documentation failure) or infant condition (informative missingness), or something else altogether. Importantly, all explanations are concerning and undermine comparative research validity.

Complete and accurate documentation is a fundamental professional obligation. If providers systematically fail to document mandated data elements, this represents a failure of professional standards and regulatory oversight. Systematic documentation failure, regardless of infant condition, indicates inadequate quality assurance, insufficient accountability, and absence of effective regulatory enforcement in out-of-hospital settings.

If missing scores disproportionately occur when infants are compromised (informative missingness), this conceals adverse outcomes. Even if only 25–50% of missing scores represent low values, risk estimates for out-of-hospital births increase substantially. The selective absence of outcome data in cases where documentation is most critical constitutes a form of reporting bias that invalidates safety comparisons.

These scenarios, and the reality likely involves elements of all, undermine the validity of safety comparisons and informed consent. Our findings demonstrate systematic documentation failure regardless of its underlying cause. The onus is on out-of-hospital providers and regulatory bodies to ensure complete reporting, not on researchers to prove what the missing data would have shown. One cannot analyze data that was never recorded. Until documentation is complete, claims of equivalent safety using only available data when many others are missing remain methodologically unreliable.

### Broader implications

In perinatal or clinical datasets informative missingness can bias analyses because the missing data pattern is not random. Informative missingness may extend beyond Apgar scores. Our data show congenital anomalies, abnormal conditions, and infant survival status were also more frequently missing in out-of-hospital births (Table 2). This warrants investigation of whether other adverse outcomes such as maternal hemorrhage, birth trauma, and transport need are similarly underreported.

The US has seen rising maternal, neonatal, and infant mortality, especially among marginalized populations, underscoring the need for accurate, complete data.^43^^,^^44^ These challenges are compounded by the absence of universal healthcare, contributing to inequities in access, variable documentation standards, and inconsistent provider accountability.^45^^,^^46^

Our findings underscore the need for methodological and regulatory reform. Professional bodies should require standardized outcome documentation. Studies with significant non-random missingness should not be cited as evidence of safety. Accurate informed consent should disclose when the evidence base is compromised. Sensitivity analyses should become standard when comparing outcomes across settings with differing data completeness.

### Documentation failure as a systematic problem

To our knowledge, this is the first national study showing that selective non-reporting of 5-min Apgar scores distorts safety assessments of US out-of-hospital births. Despite legal obligations, missing scores are disproportionately common in out-of-hospital births, suggesting systematic bias. The failure to document poor outcomes reflects selective reporting that undermines research integrity. Over 70 years after Virginia Apgar's 1953 directive that “the condition of the baby at birth be noted carefully and recorded in *every* case,” this remains unmet in many out-of-hospital births.^22^

Missing 5-min Apgar score data may result from actual adverse outcomes where documentation is delayed due to resuscitation; workflow disruptions where emergencies outpace available support; variation in training that underemphasizes standardized assessment; or deliberate omission to avoid scrutiny or liability. Such patterns are consistent with “informative missingness,” where data absence signals potential harm. We cannot exclude that some low Apgar scores were recorded as higher than actual, due to subjective interpretation, optimism bias, or implicit incentives to avoid triggering reporting protocols. This concern is underscored by strikingly elevated rates of documented Apgar scores of 10 in out-of-hospital settings, suggesting probable score inflation.^35^ However, missing documentation may also occasionally occur in cases with normal Apgar scores due to benign oversight.

Regardless of cause, systematic documentation failure invalidates comparative research. Our study answers its research question of whether mandated neonatal outcome data are documented equally across birth settings. They are not.

This finding alone has important implications: when critical outcome data are missing, the entire framework of scientific and ethical accountability collapses and comparative effectiveness research becomes invalid because outcomes that were never recorded cannot be compared. This weakens informed consent, as patients cannot make autonomous decisions without complete information. Quality improvement efforts are undermined, since one cannot improve what is not documented. Regulatory oversight fails, because accountability depends on complete, verifiable data.

### Strengths and limitations

Several limitations merit consideration. Birth certificate data may include misclassification and lack granularity on some clinical factors. Deterministic sensitivity models simulate potential bias but cannot determine the true condition of infants with missing scores. Although the assumption that missing scores reflect low values cannot be directly confirmed, this pattern is well supported by biological plausibility and empirical research.^17^, ^18^, ^19^ Studies consistently show missing data are more likely in emergency contexts, making informative missingness assumptions more plausible.^17^, ^18^, ^19^ Physicians-attended hospital births had near complete documentation of 5-min Apgar scores similar to midwife-attended hospital births.

We acknowledge geographic heterogeneity in birth practices and documentation standards across states. No state showed hospital births with consistently higher missingness than home births when sufficient data were available. The consistency of our findings, missing Apgar scores are 20–30 fold more common in–home births than hospital births in every year examined, suggests a systematic, setting-level phenomenon rather than isolated problems. State-by-state analysis shows the same pattern in all states with adequate sample sizes.

We acknowledge that missing Apgar scores may also occur in preterm births. However, we restricted analysis to term infants to avoid confounding by clinical complexity. Future research should examine missingness across gestational age strata.

While birth certificate data include other neonatal outcomes such as NICU admission and assisted ventilation, analysis of those variables was beyond this study's scope, which focuses specifically on Apgar score missingness as a sentinel indicator of documentation integrity.

We cannot establish a direct correlation between missing 5-min Apgar scores and poor neonatal outcomes in this analysis. However, the internal consistency and magnitude of observed disparities support our findings' robustness. Informative missingness is related to the outcome or variable of interest, meaning that the missingness itself carries information about the process or population being studied, and such informative missingness can bias analyses because the missing data pattern is not random.^46^ The methods applied here offer a transferable model for identifying and addressing selective outcome omissions in settings where regulatory, structural, or professional factors influence documentation. In such contexts, sensitivity analysis should become routine in comparative effectiveness research.

### Conclusions

This study highlights the critical importance of complete outcome reporting. Informative missingness in intended home births, particularly of 5-min Apgar scores, potentially obscures neonatal risk, compromises scientific validity, and misleads those interested in out-of-hospital births. Regulatory standards should not only mandate but also enforce uniform documentation of sentinel outcomes across all birth settings. Researchers should incorporate sensitivity analyses when data are incomplete, and publications should not cite studies with unaddressed non-random missingness as evidence of safety.

Most importantly, those interested in out-of-hospital births cannot exercise meaningful autonomy or provide ethically valid informed consent when critical outcome data are selectively undocumented or systematically missing. Respect for autonomy affirms the right to choose one's birth setting, but true respect for autonomy requires choices grounded in full and accurate information. Freedom of choice does not, on its own, ensure true informed consent. Therefore, decisions about out-of-hospital birth cannot be responsibly based primarily on preferences for decreased interventions when fundamental safety data are systematically missing or unreliable.

Women considering out-of-hospital birth should understand that current evidence demonstrates hospital birth is the safest setting with the most rigorous safety standards and comprehensive documentation. In the absence of reliable, complete data demonstrating the safety of United States. Home births, hospitals remain the setting with the highest standards of safety and accountability. It is not professionally defensible to represent home birth in the United States as a safe alternative to hospital births.^42^^,^^47^

## Contributors

All authors contributed substantially to the conception, design, analysis, and interpretation of the data; drafting and critical revision of the manuscript; and final approval of the version to be published. AG, RL, and FAC each contributed equally to the manuscript preparation and take responsibility for the integrity of the work.

## Data sharing statement

All data generated and analyzed in this study are openly accessible through the Centers for Disease Control natality database available at: https://wonder.cdc.gov/natality.html.

## Declaration of generative AI and AI-assisted technologies in the writing process

During the preparation of this work the author(s) used Claude and ChatGPT in order to check spelling and grammar. After using this tool/service, the author(s) reviewed and edited the content as needed and take(s) full responsibility for the content of the publication.

## Declaration of interests

The authors declare no conflicts of interest.

## References

[1] American Academy of Pediatrics Committee on Fetus and Newborn, American College of Obstetricians and Gynecologists Committee on Obstetric Practice (2015). The Apgar score. Pediatrics.

[2] Birthplace in England Collaborative Group (2011). Perinatal and maternal outcomes by planned place of birth for healthy women with low risk pregnancies: the birthplace in England national prospective cohort study. BMJ.

[3] Hollowell J., Li Y., Bunch K., Brocklehurst P. (2015). The birthplace in England national prospective cohort study: further analyses to enhance policy and service delivery decision-making for planned place of birth. Health Serv Deliv Res.

[4] de Jonge A., van der Goes B.Y., Ravelli A.C. (2009). Perinatal mortality and morbidity in a nationwide cohort of 529,688 low-risk planned home and hospital births. BJOG.

[5] Grünebaum A., McCullough L.B., Orosz B., Chervenak F.A. (2020). Neonatal mortality in the United States is related to location of birth (hospital versus home) rather than the type of birth attendant. Am J Obstet Gynecol.

[6] Wax J.R., Lucas F.L., Lamont M., Pinette M.G., Cartin A., Blackstone J. (2010). Maternal and newborn outcomes in planned home birth vs planned hospital births: a metaanalysis. Am J Obstet Gynecol.

[7] Snowden J.M., Tilden E.L., Snyder J., Quigley B., Caughey A.B., Cheng Y.W. (2015). Planned out-of-hospital birth and birth outcomes. N Engl J Med.

[8] Grünebaum A., Bornstein E., McLeod-Sordjan R. (2023). The impact of birth settings on pregnancy outcomes in the United States. Am J Obstet Gynecol.

[9] Grünebaum A., McCullough L.B., Sapra K.J. (2013). Apgar score of 0 at 5 minutes and neonatal seizures or serious neurologic dysfunction in relation to birth setting. Am J Obstet Gynecol.

[10] Grünebaum A., McCullough L.B., Sapra K.J., Brent R.L., Arabin B., Chervenak F.A. (2014). Early and total neonatal mortality in relation to birth setting in the United States, 2006–2009. Am J Obstet Gynecol.

[11] American College of Obstetricians and Gynecologists Committee on Obstetric Practice (2017). Committee opinion no. 697: planned home birth. Obstet Gynecol.

[12] Hutton E.K., Reitsma A.H., Kaufman K. (2009). Outcomes associated with planned home and planned hospital births in low-risk women attended by midwives in Ontario, Canada, 2003-2006: a retrospective cohort study. Birth.

[13] Janssen P.A., Saxell L., Page L.A. (2009). Outcomes of planned home birth with registered midwife versus planned hospital birth with midwife or physician. CMAJ.

[14] Cheyney M., Bovbjerg M., Everson C., Gordon W., Hannibal D., Vedam S. (2014). Outcomes of care for 16,924 planned home births in the United States: the midwives alliance of North America statistics project, 2004 to 2009. J Midwifery Womens Health.

[15] Hutton E.K., Reitsma A., Simioni J., Brunton G., Kaufman K. (2019). Perinatal or neonatal mortality among women who intend at the onset of labour to give birth at home compared to women of low obstetrical risk who intend to give birth in hospital: a systematic review and meta-analyses. eClinicalMedicine.

[16] Grünebaum A., Chervenak F.A. (2025). Documentation of mandated birth certificate data elements across US birth settings. JAMA Netw Open.

[17] Rubin D.B. (1976). Inference and missing data. Biometrika.

[18] Schafer J.L., Graham J.W. (2002). Missing data: our view of the state of the art. Psychol Methods.

[19] Blackwell M., Honaker J., King G. (2017). A unified approach to measurement error and missing data: overview and applications. Sociol Methods Res.

[20] Centers for Disease Control and Prevention, National Center for Health Statistics CDC WONDER: Natality public-use data 2016–2023. https://wonder.cdc.gov/natality-expanded-current.html.

[21] National Center for Health Statistics (1997). http://www.cdc.gov/nchs/data/misc/usvss.pdf.

[22] Apgar V. (1953). A proposal for a new method of evaluation of the newborn infant. Curr Res Anesth Analg.

[23] Hong J., Crawford K., Jarrett K. (2024). Five minute Apgar score and risk of neonatal mortality, severe neurological morbidity and severe non neurological morbidity in term infants—a population based cohort study. Lancet Reg Health West Pac.

[24] Moster D., Lie R.T., Markestad T. (2001). Relation between Apgar score and mortality and cerebral palsy: a population-based study of term infants in Norway. Pediatrics.

[25] Thorngren-Jerneck K., Herbst A. (2001). Low 5-minute Apgar score: a population-based register study of 1 million term births. Obstet Gynecol.

[26] Hassen T.A., Chojenta C., Egan N., Loxton D. (2021). The association between the five-minute apgar score and neurodevelopmental outcomes among children aged 8-66 months in Australia. Int J Environ Res Public Health.

[27] Iliodromiti S., Mackay D.F., Smith G.C., Pell J.P., Nelson S.M. (2016). Association between the Apgar score at 5 minutes and long-term cognitive function in childhood and adolescence: a population-based cohort study in Scotland. BMJ.

[28] Moster D., Lie R.T., Irgens L.M., Bjerkedal T., Markestad T. (2001). The association of Apgar score with subsequent death and cerebral palsy: a population-based study in term infants. N Engl J Med.

[29] Persson M., Razaz N., Tedroff K., Joseph K.S., Cnattingius S. (2018). Five and 10 minute Apgar scores and risks of cerebral palsy and epilepsy: population-based cohort study in Sweden. BMJ.

[30] Ehrenstein V. (2009). Association of Apgar scores with death and neurologic disability. Clin Epidemiol.

[31] Finster M., Wood M. (2005). The Apgar score has survived the test of time. Anesthesiology.

[32] Arnaud E., Elbattah M., Ammirati C., Dequen G., Ghazali D.A. (2023). Predictive models in emergency medicine and their missing data strategies: a systematic review. NPJ Digit Med.

[33] Agiwal V., Chaudhuri S. (2024). Methods and implications of addressing missing data in health-care research. Curr Med Issues.

[34] Jakobsen J.C., Gluud C., Wetterslev J., Winkel P. (2017). When and how should multiple imputation be used for handling missing data in randomised clinical trials – a practical guide with flowcharts. BMC Med Res Methodol.

[35] Grünebaum A., McCullough L.B., Brent R.L., Arabin B., Levene M.I., Chervenak F.A. (2015 Jul). Justified skepticism about Apgar scoring in out-of-hospital birth settings. J Perinat Med.

[36] Nethery E., Schummers L., Levine A., Caughey A.B., Souter V., Gordon W. (2021). Birth outcomes for planned home and licensed freestanding birth center births in Washington state. Obstet Gynecol.

[37] Scarf V.L., Rossiter C., Vedam S. (2018). Maternal and perinatal outcomes by planned place of birth among women with low-risk pregnancies in high-income countries: a systematic review and meta-analysis. Midwifery.

[38] Zielinski R., Ackerson K., Kane Low L. (2015). Planned home birth: benefits, risks, and opportunities. Int J Womens Health.

[39] Johnson K.C., Daviss B.A. (2005). Outcomes of planned home births with certified professional midwives: large prospective study in North America. BMJ.

[40] Rossi A.C., Prefumo F. (2018). Planned home versus planned hospital births in women at low-risk pregnancy: a systematic review with meta-analysis. Eur J Obstet Gynecol Reprod Biol.

[41] Grünebaum A., McCullough L.B., Arabin B., Brent R.L., Levene M.I., Chervenak F.A. (2016). Neonatal mortality of planned home birth in the United States in relation to professional certification of birth attendants. PLoS One.

[42] Grünebaum A., Bornstein E., Katz A., Chervenak F.A. (2022). An immutable truth: planned home births in the United States result in avoidable adverse neonatal outcomes. Am J Obstet Gynecol.

[43] The Lancet Child Adolescent Health (2021). Infant and maternal mortality in the USA. Lancet Child Adolesc Health.

[44] Joseph K.S., Lisonkova S., Boutin A. (2024). Spatiotemporal patterns and surveillance artifacts in maternal mortality in the United States: a population-based study. Lancet Reg Health Am.

[45] Taylor L.A., Coyer C., Zuckerman D. (2020).

[46] Little R.J.A., Rubin D.B. (2019).

[47] Chervenak F.A., McCullough L.B., Brent R.L., Grünebaum A. (2013). Planned home birth: the professional responsibility response. Am J Obstet Gynecol.

